# Supplementary figures and images for: Tankyrase inhibition sensitizes cells to CDK4 blockade
Source: PLoS One. 2019 Dec 31;14(12):e0226645. doi: 10.1371/journal.pone.0226645 (PMC6938305; doi:10.1371/journal.pone.0226645)

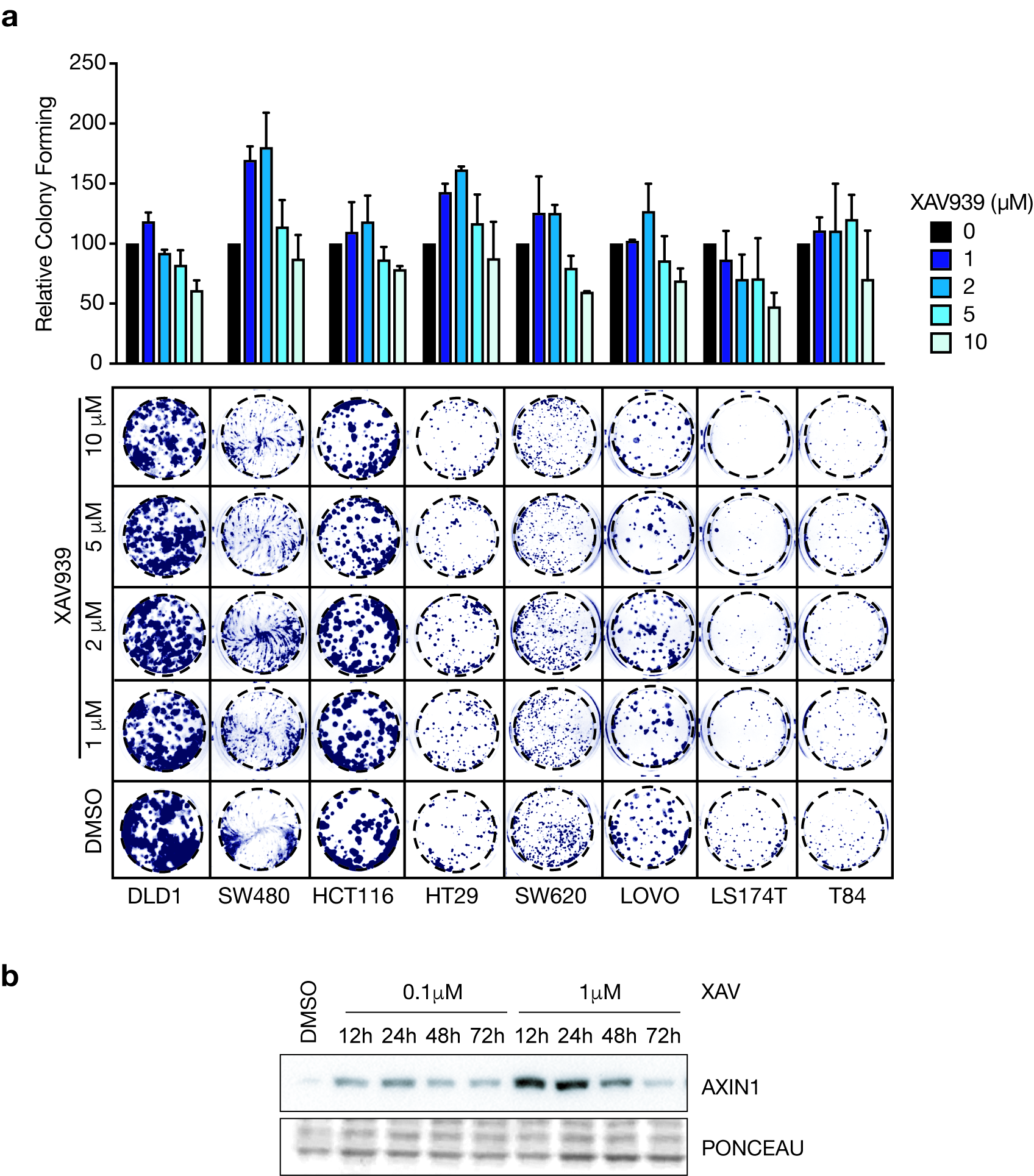

Supplement: S1 Fig — a. Colony forming assay quantification (top panel) and representative images (bottom panel) of cells grown in the indicated inhibitor concentrations (n = 2). b. Western blot detection of Axin1 accumulation at different incubation timepoints using the indicated XAV doses. (TIF) [file pone.0226645.s001.tif]

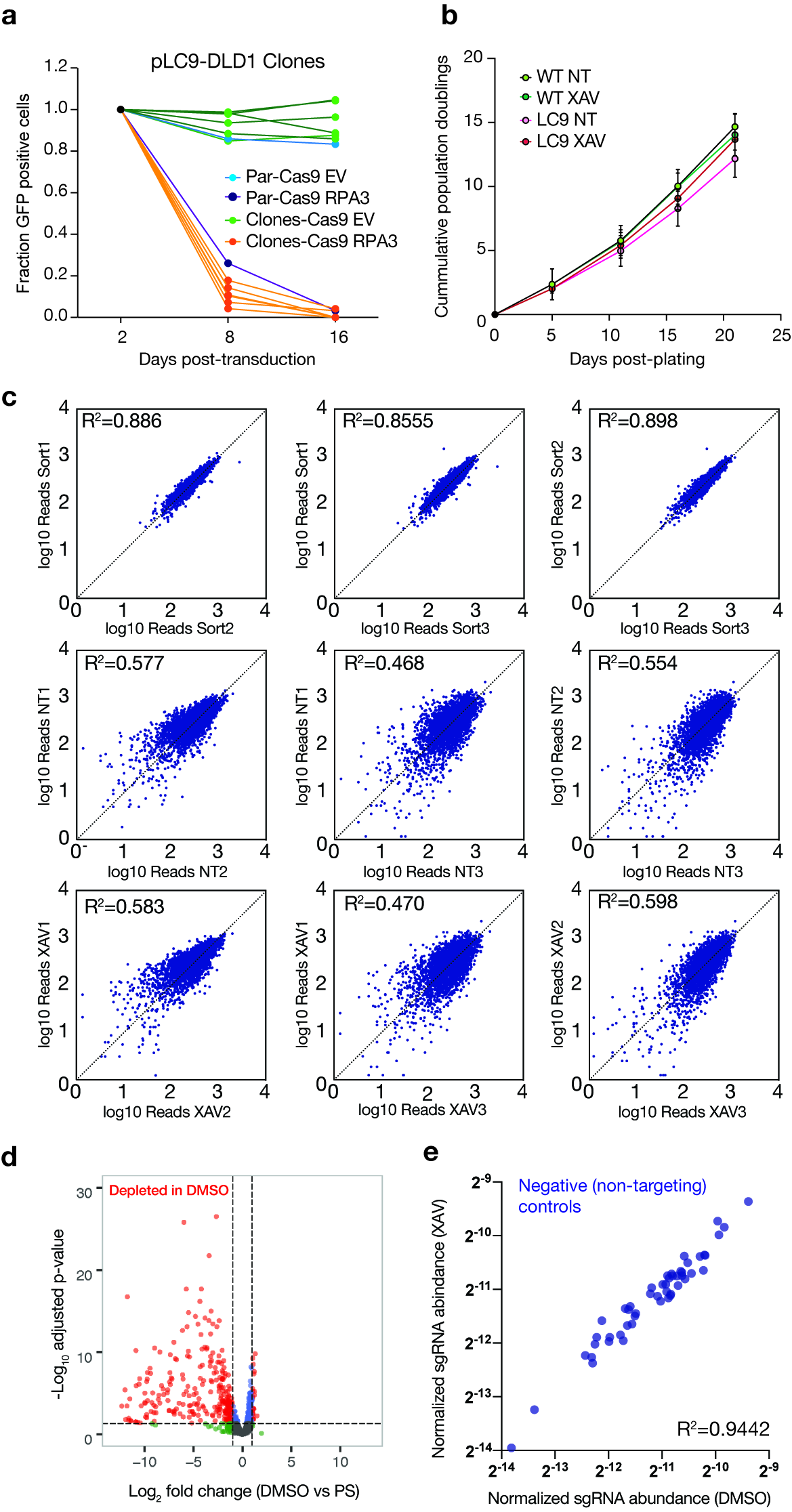

Supplement: S2 Fig — a. Fluorescence competition assays of DLD1 clones stably expressing pLenti-Cas9 and further transduced with RPA3 gRNA or a control gRNA empty vector. b. Cell growth by cumulative population doubling estimation in DLD1 cells treated as indicated with 1mM XAV or DMSO (n = 3). c. Dot plots of the gRNA reads in two-by-two comparisons. Correlation coefficients (R2) across conditions are shown. d. Volcano plot of Log10 adjusted p-values vs log2 fold-change in gRNA representation in DMSO D17 vs post-sorting cells (D3, PS). In red, gRNAs significantly depleted in DMSO. e. Normalized control (non-targeting) gRNA counts in DMSO (D17) vs XAV (D17)-treated cells. (TIF) [file pone.0226645.s002.tif]

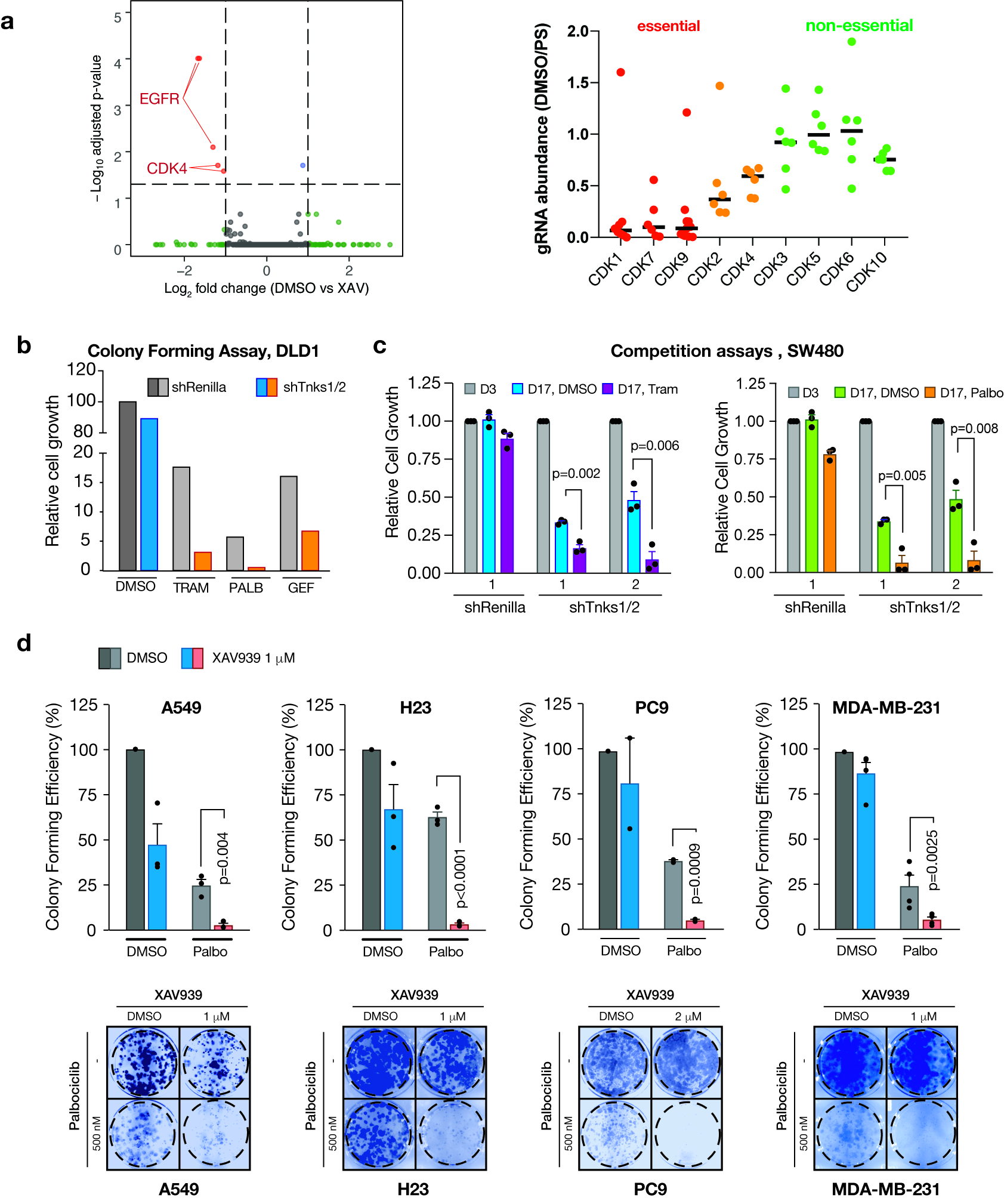

Supplement: S3 Fig — a. Left panel, dot plot showing log10 Adj p values and log2 fold change of DMSO (D17) vs XAV (D17) in DLD1 cells. Significantly represented gRNAs are highlighted in red. Right panel, relative gRNA abundance of human CDK protein members at D17 (DMSO) vs post-sorting (PS) samples. b. Quantification of the colony forming assay shown in Fig 2F. DLD1 cells stably expressing inducible shRNAs against TNKS were treated with the indicated drugs +/- dox. c. Fluorescent competition assays in SW480 cells stably expressing shRNAs against TNKS1/2, treated with Trametinib (left) or Palbociclib (right) +/- dox. The GFP positive cells represent the proportion of shRNA-expressing fraction of each population, relative to D2 post-transduction. d. Colony forming assays (bottom panels) and quantification (top panels) of XAV and Palbociclib combinations as indicated, for a panel of epithelial cells lines, including lung and breast. N = 2–3 independent experiments, and p values represent Student’s t test. (TIF) [file pone.0226645.s003.tif]

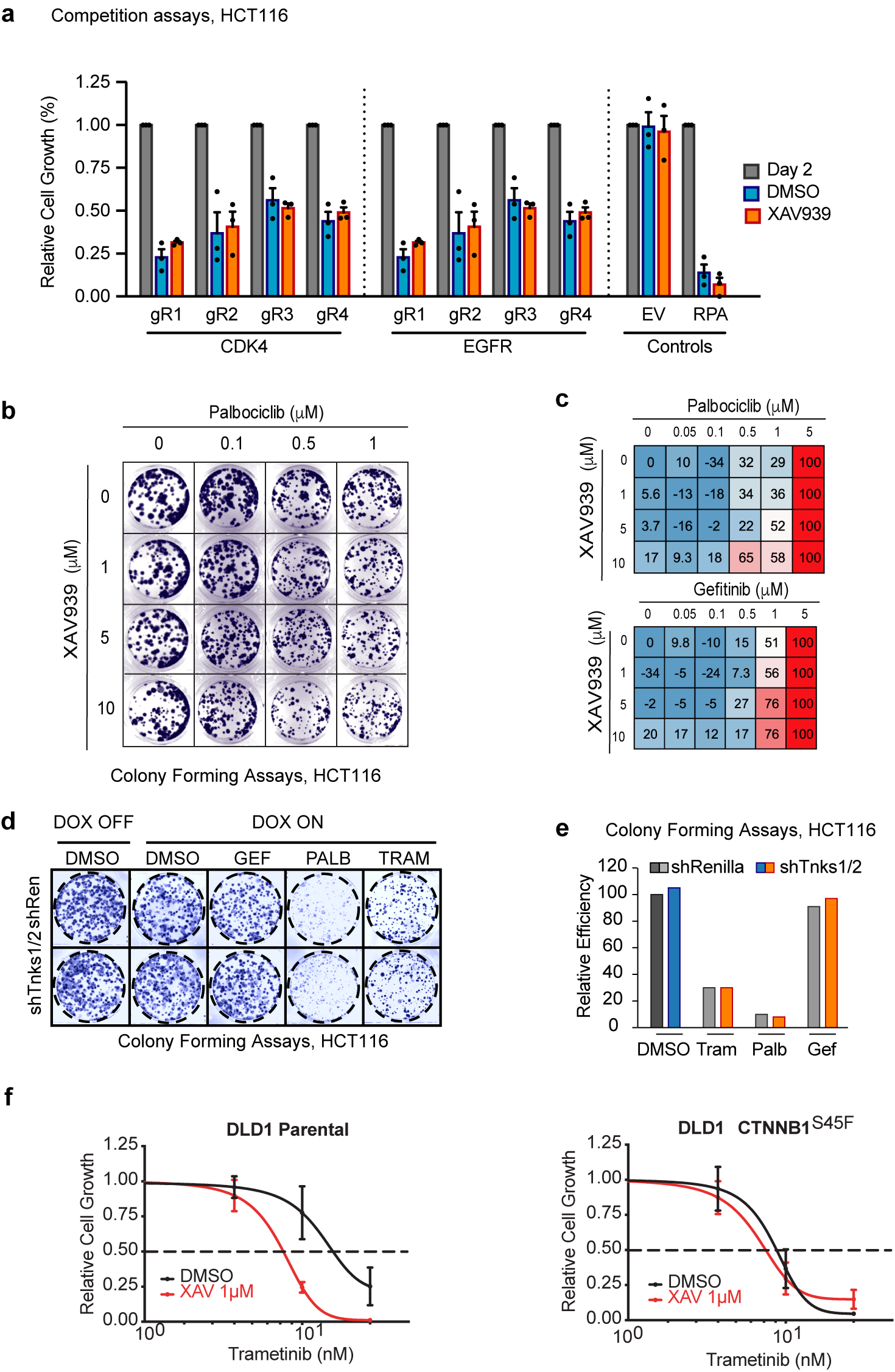

Supplement: S4 Fig — a. Fluorescent competition assay in HCT116 clones stably expressing Cas9 and further transduced with the indicated gRNAs, in the presence or absence of XAV. N = 3 clones, Student’s t test b. HCT116 cells treated with the indicated doses of XAV and Palbociclib were seeded in colony-forming assays. c. Quantification of cell proliferation inhibition in HCT116 cells treated with the indicated concentrations of Palbociclib or Gefitinib +/-XAV. d. shRNA-mediated knock-down of TNKS does not influence cellular sensitivity to Palbociclib, Trametinib or Gefitinib in HCT116 cells. e. Quantification of the experiment shown in (d). f. Trametinib sensitivity in DLD1 parental or base-editing-generated S45F mutant isogenic DLD1 cell lines. (TIF) [file pone.0226645.s004.tif]
